# MuSK EAMG: Immunological Characterization and Suppression by Induction of Oral Tolerance

**DOI:** 10.3389/fimmu.2020.00403

**Published:** 2020-03-17

**Authors:** Debby Reuveni, Revital Aricha, Miriam C. Souroujon, Sara Fuchs

**Affiliations:** ^1^Department of Immunology, Weizmann Institute of Science, Rehovot, Israel; ^2^Department of Natural Sciences, The Open University of Israel, Ra'anana, Israel

**Keywords:** myasthenia gravis, muscle-specific receptor tyrosine kinase (MuSK), neuromuscular junction (NMJ), oral tolerance, T regulatory cells

## Abstract

Myasthenia gravis (MG) with antibodies to the muscle-specific receptor tyrosine kinase (MuSK) is a distinct sub-group of MG, affecting 5–8% of all MG patients. MuSK, a receptor tyrosine kinase, is expressed at the neuromuscular junctions (NMJs) from the earliest stages of synaptogenesis and plays a crucial role in the development and maintenance of the NMJ. MuSK-MG patients are more severely affected and more refractory to treatments currently used for MG. Most patients require long-term immunosuppression, stressing the need for improved treatments. Ideally, preferred treatments should specifically delete the antigen-specific autoimmune response, without affecting the entire immune system. Mucosal tolerance, induced by oral or nasal administration of an auto-antigen through the mucosal system, resulting in an antigen-specific immunological systemic hyporesponsiveness, might be considered as a treatment of choice for MuSK-MG. In the present study we have characterized several immunological parameters of murine MuSK-EAMG and have employed induction of oral tolerance in mouse MuSK-EAMG, by feeding with a recombinant MuSK protein one week before disease induction. Such a treatment has been shown to attenuate MuSK-EAMG. Both induction and progression of disease were ameliorated following oral treatment with the recombinant MuSK fragment, as indicated by lower clinical scores and lower anti-MuSK antibody titers.

## Introduction

Myasthenia gravis (MG) is an autoimmune disease characterized by skeletal muscle weakness as a result of an immunological attack at the neuromuscular junction (NMJ). In 5–8% of MG patients the autoantibodies present in the sera are against the muscle-specific receptor tyrosine kinase (MuSK) classifying these patients as a distinct MG sub-group called MuSK-MG (1). MuSK is a tyrosine kinase receptor expressed from early stages of synaptogenesis at the NMJ and has been shown to play a critical role in NMJ development and maintenance (2). Low-density lipoprotein receptor-related protein 4 (LRP4) and MuSK act together as a receptor for Agrin, a motor-neuron-derived matrix proteoglycan. Agrin binding results in dimerization of MuSK and LRP4 followed by activation of MuSK (3). However, it is still unknown how these molecules regulate NMJ formation (4).

The use of currently immunosuppressive therapies for MuSK-MG patients is challenging as patients are prone to develop severe facial weakness and bulbar symptoms, including dysphagia, dysarthria and respiratory crisis with some atrophy of facial muscles making the treatment less effective (5, 6). Thymus alterations are common in Acetylcholine receptor (AChR)-MG patients whereas in MuSK-MG patients, thymus histology is mostly normal-for-age, with scattered lymphoid infiltrates (7). Among the treatments available, the use of acetyl-cholinesterase inhibitors is unsatisfactory and thymectomy does not improve the course of disease (1). Rituximab, a B cell depleting agent, was recently shown to benefit patients in uncontrolled studies; yet, data from controlled prospective studies on the use of rituximab in MuSK-MG patients are not available, thus, leaving immunosuppression as the mainstay of treatment (8). The severe form of MuSK-MG requires emergent and aggressive treatment to manage respiratory distress. A marked improvement in disease symptoms is achieved by corticosteroids but disease flares are frequent during dosage tapering, as a result the patients depend on treatment thus, it is crucial to develop improved and better treatment modalities.

To date, general immunosupression is the mainstay treatment for autoimmune diseases. The main challenge for immunologists is to develop novel treatments that will manipulate specifically or correct the abnormal immune response leaving the overall immune response intact.

Specific systemic tolerance to an antigen can be achieved by exposing mucosal surfaces to a particular antigen (9), and it is now accepted that it plays a crucial role in preventing disorders such autoimmunity and food allergies. While the process is not fully understood, recent years have seen a number of important advances due to expansion of knowledge in cellular immunology. One of the most important developments in the field has been the realization that the microbiota has dramatic effects on immune function throughout the body. It encouraged scientists to modulate many experimental autoimmune diseases by induction of mucosal tolerance to a specific autoantigen (10).

We have shown in our previous studies that mucosal (oral or nasal) administration of torpedo AChR prior to disease induction resulted in EAMG suppression and was accompanied by inhibition of the humoral response as well as cellular responses to AChR (11, 12). Moreover, EAMG clinical manifestation was suppressed when the antigen was administered during the acute phase of the disease (13).

Shigemoto et al. have developed a MuSK-EAMG model (14) in which FVB/N complement-deficient mice are immunized with a recombinant MuSK protein and a month after 100% of these mice synchronously develop MuSK-EAMG. The use of this model is particularly useful for the development and testing of novel therapeutic strategies as disease progression is predictable and the model resembles well the human disease.

In this study we have induced the MUSK-EAMG experimental model disease in mice and characterized some of its immunological properties in order to apply it as a model for therapeutic experiments. Specifically, we report on our successful efforts to induce mucosal tolerance to the MuSK antigen by oral application of the recombinant extracellular domain of the MuSK protein.

## Materials and Methods

### Animals

Female FVB/N mice aged 6-8 weeks were obtained from the Animal Breeding Center of The Weizmann Institute of Science, Rehovot, Israel and were maintained at the Institute's animal facilities. All the experiments in this study were performed according to the institutional guidelines for animal care.

### Production of Recombinant Rat MuSK

pCEP-PU vector containing the His-tagged extracellular domain of recombinant rat MuSK [aa 21-491; (15)] was kindly provided by A.R. Punga (Upsala, Sweden). The plasmid was transfected, using Lipofectamine 2000 from Invitrogen (Carlsbad, CA) into HEK 293 EBNA cells. Large-scale production of the recombinant protein was performed at the Proteomics Unit of the Weizmann Institute. The recombinant MuSK protein was produced by the mammalian cells and was secreted to the medium under serum free conditions. Cell supernatant was subjected to a Ni-NTA super-flow column (Qiagen, Hilden, Germany) for protein purification. The purity of the protein obtained following Ni affinity chromatography and gel filtration was ~95%. Concentration was determined at OD 280 nm.

### Induction and Clinical Evaluation of MuSK-EAMG

On day 0, adult female mice were anesthetized (Ketamine: 111 mg/kg and Xylazine: 22 mg/kg) and immunized subcutaneously, each with a total volume of 200 μl of recombinant MuSK (20 or 40 μg/mouse), emulsified in complete Freund's adjuvant (CFA) from Sigma-Aldrich (St. Louis, MO), as follows: 20 μl in each hind foot pads, 40 μl at the base of the tail and 20 μl in each of 6 well-separated sites on the back. On day 14 post-injection, all mice were boosted by 6 well-separated sites on the back with 20–40 μg MuSK emulsified with incomplete Freund's adjuvant (IFA) from Sigma-Aldrich (St. Louis, MO). Control mice were immunized by CFA and IFA only.

Mice were observed, weighed and scored blindly on alternate days, for the clinical severity of disease as follows: 0 - Healthy mouse; 1 - Body weight loss; 2 - Body weight loss, weakness, prominent cervicothoracic hump; 3 - Body weight loss, weakness, prominent cervicothoracic hump, tremor and ungroomed fur; 4 - Dead.

The method of blinding employed in our experiments was as follows: FVB/N mice were randomly divided into experimental groups: MuSK and CFA. One operator was assigned to the experimental treatments (MuSK immunization) while a second person who assessed the mice (weight, scoring) remained blinded to the experimental groups until the end of the experiment.

All experimental groups consisted of 10 mice each, unless otherwise specified and all experiments were repeated 2–3 times.

### Anti-MuSK Antibodies

Sera of treated mice were collected by retro-orbital bleeding 6 weeks following disease induction. The levels of anti-MuSK antibodies were determined by standard ELISA as follows: Microtiter plates were coated with recombinant MuSK protein (10 mg/100 ml in Tris-Cl, pH 8.0), and reacted with 100 μl of the tested mouse serum, at a dilution of 1:1000 for total IgG. Rabbit anti-Mouse alkaline phosphatase antibody (1:10000 Jackson, immunoresearch laboratories; West Grove, PA) was added followed by alkaline phosphatase-conjugated streptavidin. Antibody levels were evaluated by measuring the optical density at 405 nm.

### Immunofluorescence Flow Cytometry

Flow cytometry analysis was performed on splenocytes of MuSK-immunized mouse and of control mouse. Spleen cells were suspended in FACS wash buffer (PBS, 5% BSA) and incubated for 60 min at 4°C in the dark with antibodies to the tested cell surface molecules. Cells were washed and analyzed on a FACScan flow cytometer. The following antibodies were used for flow cytometry: FITC-conjugated anti-mouse CD4 (L3T4) and APC-conjugated anti-mouse CD25 (PC61.5). For intracellular staining, cells were fixed and permeabilized using the fixation/permeabilization kit from e-Bioscience (San Diego, CA) followed by staining with PE-conjugated anti-FoxP3 antibody (e-Bioscience).

### RNA Isolation and Quantitative Real-Time PCR

Total RNA was extracted from mice splenocytes and muscles at the end of the experiment (8 weeks following immunization with MuSK). Extraction was performed using the high pure RNA Isolation Kit (Roche, Mannheim, Germany) according to the manufacturer's instructions. The concentration of total RNA was measured by NanoDrop ND-1000 Spectrophotometer (Thermo Scientific, Wilmington, DE). Complementary DNA was prepared and quantitative real-time reverse transcription (PCR) was performed using the LightCycler system (Roche) according to the manufacturer's instructions. Primer sequences (forward and reverse, respectively) are given in Table 1.

**Table 1:** Primer sequences.

**Gene**	**Forward primer**	**Reverse primer**
MuSK	5′-GGCCGTGTAAGACCAG-3′	5′-GGAACGTAACCGGGAT-3′
TGFβ	5′-CAAGGGCTACCAT GCCAACT-3′	5′-CCGGGTTGTGTTGGTTGTAGA-3′
Foxp3	5′-TGCTCCATACCTTGAACAC-3′	5′-CACTATATAGTCACCCCAAC-3′
Cathepsin-l	5′-GTTCTGGTGGTTGGCT-3′	5′-GTAGTGTCCGTAAGTCCT-3′
IL-18	5′-TCCCAGACCAGACTGATAA-3′	5′-CTGGCACACGTTTCTGA-3′
IL-15	5′-CTGGCACACGTTTCTGA-3′	5′-CAGCAGGTGGAGGTACCTTAA-3′
β-actin	5′-TACTGCCCTGGCTCCTAGCA-3′	5′-TGGACAGTGAGGCCAGGATAG-3′.

*β-actin was used as the house-keeping gene*.

### MuSK-Specific Oral Tolerance Induction

Musk-specific oral tolerance was attempted by feeding of FVB/N mice with recombinant MuSK, essentially as described by us for the induction of oral tolerance of AChR-EAMG by a recombinant AChR fragment (11). FVB/N mice were fed, by means of a feeding tube, each with either recombinant MuSK (120 μg/100 μl/mouse) or with a control irrelevant protein (Ovalbumin, Sigma), both diluted in PBS. Treatment was initiated one week before disease induction and continued 3 times a week, until the end of the experiment.

Disease evaluation was performed as mentioned above: FVB/N mice were randomly divided into experimental groups: MuSK and OVA oral administration. One operator was assigned to the experimental treatments (feeding by gavage and MuSK immunization) while a second person who assessed the mice (weight, scoring) remained blinded to the experimental groups until the end of the experiment.

### Statistics

The results are presented as mean ± SEM. Two-way ANOVA test was used to compare disease assessment vs. control and treatment vs. control groups all along the experiment. Differences in mean values were compared between treatment and control groups by the Student's *t*-test. *p* values < 0.05 were considered as significant.

## Results

### Induction of MuSK-EAMG

**Figure 1:**
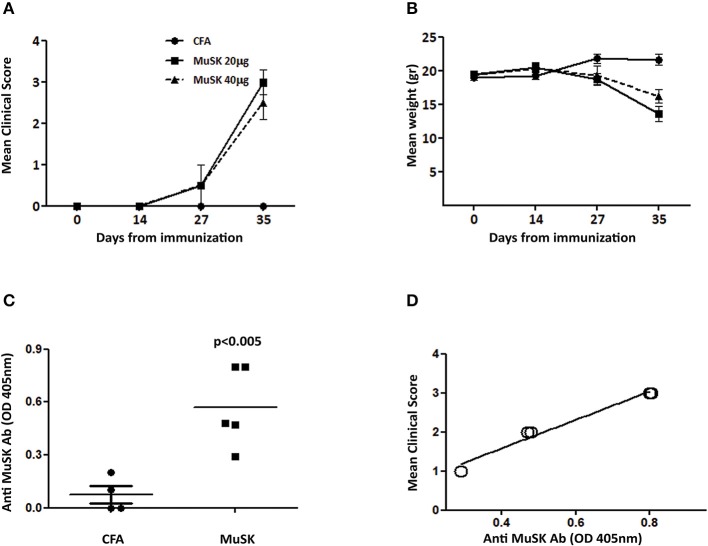
Clinical characterization and antibody titers in MuSK-EAMG, induced in FVB/N mice. FVB/N female mice were immunized twice with 20–40 μg (as indicated) of recombinant MuSK in CFA, or with CFA alone, as a control (*n* = 6). Mice were followed up for clinical score **(A)** and weight loss changes **(B)**. Anti-MuSK antibody titer was tested 4 weeks following immunization, by ELISA **(C)**, and correlation with disease severity was tested **(D)**. *P* < 0.001 in **(A,B)**. Analyzed by the two-way ANOVA test.

MuSK-EAMG, an experimental model of MuSK-MG, has been established in our lab in FVB/N female mice, according to Mori et al. (14), which observed that these mice are highly susceptible to MuSK-EAMG induction. 8 weeks old female FVB/N mice were immunized with recombinant MuSK protein (20 or 40 μg/mouse, as indicated) in CFA on day 0 and boosted 14 days later, with a similar dose of antigen, in incomplete Freund's adjuvant (IFA). All immunized mice manifested disease symptoms including severe muscle weakness and tremors within 2 weeks from the second injection. At the end of the experiment (35 days after disease induction) the CFA control group had a clinical score of 0, the MuSK 20 μg had a clinical score of 3 ± 0.6 (SD), and the MuSK 40 μg had a clinical score of 2.5 ± 1 (SD) (Figure 1A). These symptoms were observed synchronously in all animals, along with the appearance of a prominent cervicothoracic hump, indicating weak cervical extensor muscles and ungroomed fur. In addition, it should be noted that the MuSK-injected mice exhibited weight loss, corresponding to the progression of disease (Figure 1B). In contrast, control mice injected with PBS in CFA did not exhibit weight loss or any symptoms of disease (Figures 1A,B). Similar disease severity and antibody levels were observed following immunization with either 20 or 40 μg/mouse (Figure 1A) and for further experiments we have used 20 μg of MuSK for disease induction.

### Anti-MuSK Antibody Titers Correlate With Disease Severity

Anti-MuSK IgG antibodies were analyzed by ELISA and were detected in all MuSK-immunized mice, whereas control CFA-immunized mice had no detectable antibodies to MuSK (Figure 1C). Interestingly, in contrast to AChR-EAMG, in which disease severity has no correlation to the levels of anti-AChR autoantibody titers, in MuSK EAMG - there seems to be a good correlation between anti-MuSK antibody and disease severity (Figure 1D). Such a correlation has been also observed and reported in MuSK-MG patients (5).

### MuSK- Immunized Mice Show Specific Muscle Damage

In order to test whether the induction of MuSK-EAMG results in muscle damage, the mRNA expression of several genes was examined in samples derived from masseter muscles from sick (MuSK-immunized) and control mice.

**Figure 2:**
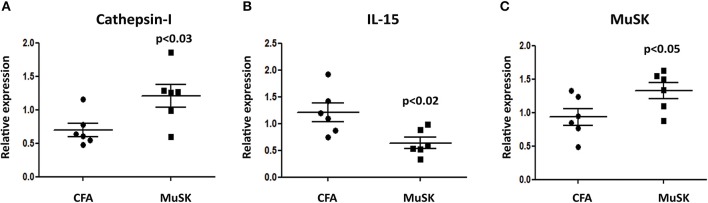
MuSK immunized mice exhibit specific muscle damage. FVB/N mice were sacrificed 5 weeks after disease induction and RNA was isolated from masseter muscles. The expression levels of cathepsin-l **(A)**, IL-15 **(B)** and MuSK **(C)** were analyzed by quantitative real time RT-PCR and compared to the levels obtained in CFA-immunized control mice. β-actin was used as an internal control for normalization. All data are presented as mean ± SEM. Unpaired Student *t* test was employed. Representative out of two experiments (*n* = 6).

The initiation of protein degradation involved among others the lysosomal endopeptidase enzyme Cathepsin l. We have observed that the level of cathepsin 1 mRNA expression is significantly increased in MuSK-immunized mice, as depicted in Figure 2A, indicating muscle damage in sick mice. Likewise, there is also a significant increase in the expression of MuSK in MuSK-immunized mice, probably as a compensatory mechanism. In addition, IL-15, which is highly expressed in skeletal muscle and is believed to be a myokine, improve muscle glucose homeostasis and oxidative metabolism, was decreased in MuSK immunized mice (Figures 2B,C, respectively).

### Treg Cell Frequency Is Decreased in MuSK-EAMG Mice

**Figure 3:**
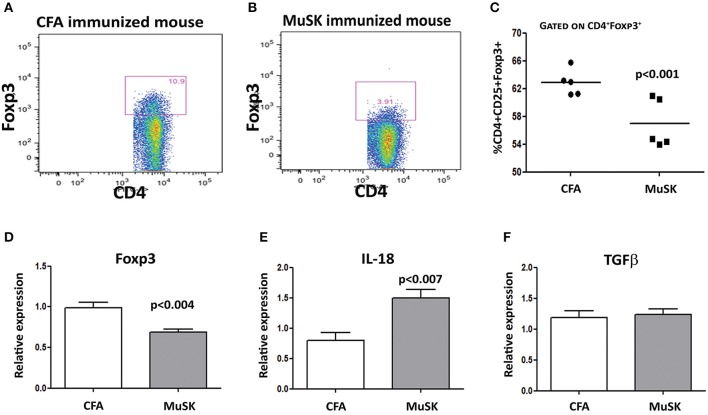
Decreased Treg cell frequencies in spleens of MuSK-EAMG mice immunized mice. Spleens from control and MuSK immunized mice were harvested at the end of the experiments and analyzed by Flow cytometry and rt-PCR analyses. Representative Flow cytometry analysis of control immunized mouse **(A)** and MuSK immunized mouse **(B)**. Graphical summary of the frequency of CD4+CD25+FoxP3+ cells **(C)**. Expression levels of FoxP3, IL-18 and TGF-β (**D–F**, respectively) were evaluated by RT-PCR. β-actin was used as an inner control for normalization. All data are presented as mean ± SEM. Unpaired Student *t* test has been employed. Representative out of two experiments (*n* = 12).

We have analyzed the T cell subpopulations of MuSK-immunized mice, and of control, adjuvant-immunized mice. FACS analyses were performed on spleen cells, 4 weeks after disease induction. To test whether there are changes in the frequency of CD4^+^CD25^+^Foxp3^+^ Treg cells in MuSK-EAMG mice, their splenocytes were stained for CD4, CD25 and Foxp3, by specific antibodies. As shown in Figure 3, the percentage of CD4+CD25+Foxp3^+^ cells among CD4^+^CD25^+^ cells, in the spleens of MuSK-EAMG mice (Figures 3B,C) is lower when compared to the percentage of such cells from healthy adjuvant-immunized controls (Figures 3A,C) These findings suggest that alterations in the Treg cell population may be involved in the immunopathology of MuSK-MG. Furthermore, we have observed a significant decrease in FoxP3 mRNA expression (Figure 3D) in MuSK immunized mice, as compared to control CFA-immunized mice. The decreased expression of FoxP3 in MuSK- immunized mice is in agreement with the flow cytometry results (Figures 3A–C), supporting a reduced frequency of Treg cells, as a result of the induced disease. In addition, we have also observed elevated levels of IL-18 mRNA (Figure 3E) whereas the expression levels of TGFβ were not changed in MuSK immunized mice when compared to CFA immunized mice.

### Suppression of MuSK-EAMG Following Induction of Oral Tolerance to MuSK Protein

Toward developing an antigen-specific treatment for MuSK-MG, we have attempted to develop an oral tolerance approach by feeding with recombinant MuSK-protein, by a similar protocol employed by us previously (11, 12) oral tolerance induction in AChR-EAMG. For these experiments we have first prepared large amounts of recombinant rat-MuSK protein, as described above in the Materials and Methods section. Preliminary experiments indicated that a dose of 120 μg recombinant MuSK/dose/mouse was optimal (Data not shown).

The oral tolerance experiment was initiated one week before disease induction and continued for 3 times a week, until the end of the experiment. Clinical scores and weights were evaluated, blinded, 3 times a week in 3 different experiments; each experimental group consisted of 10 mice.

**Figure 4:**
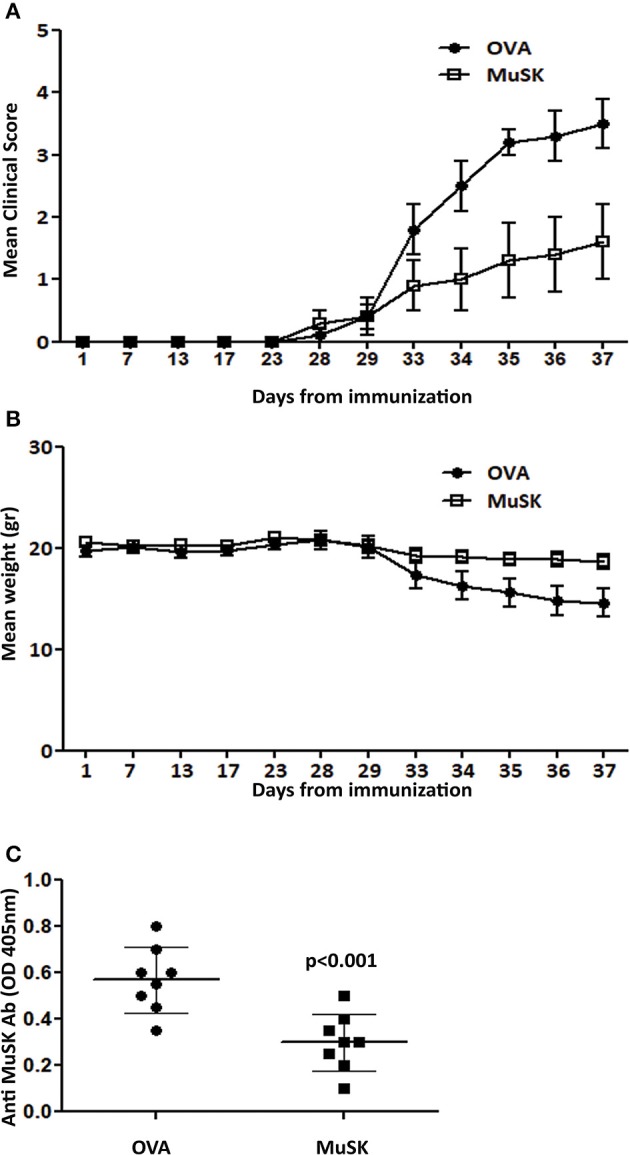
Oral treatment suppresses MuSK-MG. Mice were fed with recombinant MuSK protein or OVA protein as a control, 3 times a week, starting one week before MuSK immunization and until the end of the experiment. Mean clinical score **(A)** and weight changes **(B)** in mice fed with 120μg MuSK or OVA/mouse/dose. Total MuSK specific IgG, tested by ELISA **(C)**. *n* ≥ 8 mice for each group, in 3 different experiments. *P* < 0.001 in **(A,B)**. Analyzed by the two-way ANOVA test. Representative out of three experiments.

Administration of recombinant rat MuSK protein resulted in a significant therapeutic effect in MuSK- treated mice, accompanied also by monitoring the levels of weight changes, as depicted in Figures 4A,B, respectively. Thus, the severity of disease was significantly lower in the group of mice fed with recombinant MuSK than in the control group of mice (Figure 4A), whereas, the weight loss was less pronounced with disease progression, in the MuSK-fed group (Figure 4B).

Concomitantly with the effect of oral tolerance induction on MuSK-EAMG progression, a suppressive effect on MuSK-specific IgG antibody has also been observed. Thus, the titers of total MuSK-specific IgG in the sera of mice that were fed with MuSK, were lower than the titers in mice in the control OVA-fed mice (Figure 4C).

### The Effect of Oral Tolerance Induction on Cytokine Profile

**Figure 5:**
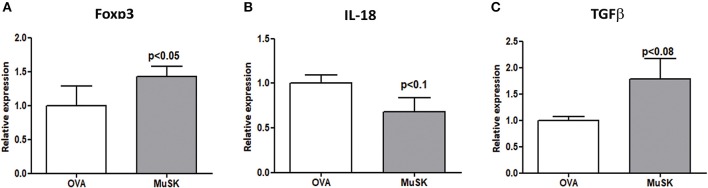
MuSK-fed mice display increased levels of Tregs associated genes. Spleens were harvested at the end of the experiment and the RNA was isolated. Expression levels of Foxp3, IL-18, and TGF-β (**A–C**, respectively) were evaluated by RT-PCR. β-actin was used as an inner control for normalization. All data are presented as mean ± SEM (*n* ≥ 5). Unpaired Student *t* test has been employed.

In light of our previous results showing the alterations in the Treg sub-population in MuSK-immunized mice, we decided to evaluate the effect of oral tolerance treatment on Treg associated genes by RT-PCR from spleens of MuSK and OVA fed mice. As shown in Figure 5, mice that were orally treated by recombinant MuSK had significantly increased expression levels of Foxp3 and TGF-β, which are essential for Treg induction, activation and maintenance when compared to OVA-fed mice. A marked decrease in IL-18 expression was observed in MuSK-fed mice, as compared to OVA-fed mice. These results imply that oral treatment may have a protective effect on MuSK immunized mice perhaps by induction of tolerance rather than by anergy induction of T-cells.

## Discussion

The present study was aimed to characterize immunological parameters of MuSK EAMG and to assess selective therapeutic strategy for MuSK myasthenia gravis.

We utilized a recombinant MuSK protein for immunization and showed that 100% of mice developed MuSK myasthenia gravis, which was synchronously developed within a month after immunization. MuSK immunized mice manifested human disease symptoms, including severe muscle weakness. This could relate to cathepsin-l up-regulation in the muscles of MuSK immunized mice, an endopeptidase that participates in pathological responses leading to muscle loss. Cathepsin-l was also shown to be up-regulated in the muscles of rats immunized with torpedo AChR (16). Additionally, MuSK immunized mice had specific anti-MuSK antibodies, which correlated with disease severity. This was in agreement with other reports that showed that serum anti-MuSK antibodies from patients correlated with clinical symptoms and response to immunotherapy. In a clinical study, the clinical score and disease classification correlated with anti-MuSK antibody distribution in 83 samples from 40 patients, and treatment with immunosuppressive agents resulted in a significant decrease in the MuSK IgG levels in individual patients (17).

The pro-inflammatory cytokine IL-18 was shown to be involved in the production of IFN-γ as well as the production of IL-12 that shifts the immune response toward a Th-1 phenotype. It was shown that IL-18 plays a role in the pathogenesis of many diseases. IL-18 knockout mice were resistant to EAMG induction (18). In our model, MuSK immunized mice manifested elevated expression levels of IL-18, indicating an inflammatory response upon MuSK immunization that may result in a dominant Th1 response.

Homeostasis and self-tolerance is achieved mainly by immune regulatory T (Treg) cells. Dysfunction and/or altered Treg cell numbers can result in the development of autoimmune diseases (19). Animal models revealed that development of autoimmunity was due to defects in the CD4+CD25+FoxP3+ Treg cell population (20). Treg cells impairment is evident in several autoimmune diseases such as type 1 diabetes, multiple sclerosis, systemic lupus erythematosus (SLE), rheumatoid arthritis, inflammatory bowel disease (IBD), autoimmune hepatitis and psoriasis (21, 22). Although there is no consensus on decreased percentage of Treg cells in MG patients, including MuSK-MG, many studies report that these cells have reduced suppressive activity. (23, 24) but there is no evidence to their role in MuSK myasthenia gravis. Our results demonstrated dysregulation of CD4^+^CD25^+^Foxp3^+^ Treg cells in MuSK-EAMG mice; the percentage of CD4+CD25+Foxp3^+^ out of CD4^+^ cells in the spleen of MuSK-EAMG mice is reduced compared to healthy adjuvant-immunized controls, as was also for Foxp3 mRNA expression.

Induction of oral tolerance involves many mechanisms that modulate the immune response against auto antigens. Oral tolerance has an impact on the numbers and function of Treg cells, on the secretion of pro and anti-inflammatory cytokines and on Th-1/Th-2 effector cells (25). This observation led to attempts to regulate many autoimmune diseases by induction of mucosal tolerance to auto antigens (10, 26–28). We have previously shown that EAMG was prevented in rats by oral or nasal administration of AChR-derived recombinant fragments when treatment started prior immunization, moreover, ongoing disease was suppressed when mucosal tolerance treatment started at the acute or chronic phase of the disease (11, 29). Mucosal tolerance induction resulted in a marked decrease in AChR specific T cell proliferative response and IL-2 production in addition to reduced levels of AChR auto-antibodies titers. The immune response shifted toward Th-2/Th-3 in addition to down-regulation of co-stimulatory factors. The underlying mechanism for the mucosal tolerance induced by the AChR fragments was shown to be active suppression and not clonal anergy.

Here we demonstrate for the first time an attempt to modulate MuSK myasthenia gravis by feeding mice with low doses of recombinant MuSK. The immune response acts differently to the dose of the antigen administered. High doses of antigens leads to anergy or apoptosis of antigen specific immune cells while administration of low doses of antigen leads to induction of antigen specific Tregs (30–32).

Administration of recombinant MuSK protein resulted in a significant therapeutic effect in treated mice, accompanied by a corresponding effect in weight loss. Additionally, the titer of total MuSK specific IgG in the serum at the end of the experiment was lower in MuSK fed mice, indicating amelioration of the disease. Moreover, expression level of Foxp3 and TGFβ were elevated in MuSK fed-mice suggesting that oral administration of MuSK modulated Tregs.

Taken together, our results demonstrated oral tolerance efficacy in MuSK-EAMG model. This therapeutic potential should be further explored and considered as a novel approach for MuSK-MG treatment and hopefully be effective and safe for the benefit of MuSK-MG patients.

## Data Availability Statement

All datasets generated for this study are included in the article/supplementary material.

## Ethics Statement

The animal study was reviewed and approved by the Weizmann Institutional Animal Care and Use Committee.

## Author Contributions

DR, SF, and MS designed and guided the research. DR and RA performed the animal experiments and analyzed most of the experiments. DR and SF wrote the manuscript. All authors reviewed and approved the manuscript.

### Conflict of Interest

The authors declare that the research was conducted in the absence of any commercial or financial relationships that could be construed as a potential conflict of interest.

## Abbreviations

MG: Myasthenia gravis
MuSK: Muscle specific tyrosine kinase
NMJ: Neural muscular junction
AChR: Acetylcholine receptor
EAMG: experimental autoimmune myasthenia gravis
CFA: complete Freund's adjuvant
IFA: Incomplete Freund's adjuvant
TGFβ: transforming growth factor beta
Foxp3: Forkhead box P3
IL-18: Interleukin 18
IL-15: Interleukin 15
OVA, Ovalbumin protein and Tregs: T regulatory cells.
